# G‐Protein‐Coupled Receptor 84 Aggravates Early Brain Injury via Microglial NLRP3‐ASC Inflammasome After Subarachnoid Hemorrhage

**DOI:** 10.1002/brb3.70379

**Published:** 2025-04-09

**Authors:** Kun Jiang, Yan Zou, Yue Song, Long'jiang Zhou, Bing'tao Zhang, Xiao'ming Zhou, Xin Zhang

**Affiliations:** ^1^ Department of Neurosurgery, Jinling Hospital, Jinling School of Clinical Medicine Nanjing Medical University Nanjing China; ^2^ Department of Neurosurgery The Affiliated Huaian No. 1 People's Hospital of Nanjing Medical University Huaian China; ^3^ Nanjing Jinling Hospital Affiliated Hospital of Medical School, Nanjing University Nanjing China; ^4^ Department of Neurology The Affiliated Hospital of Yangzhou University, Yangzhou University Yangzhou China

**Keywords:** G‐protein‐coupled receptor 84, NLRP3 inflammasome, pyroptosis, subarachnoid hemorrhage

## Abstract

**Background:**

Subarachnoid hemorrhage (SAH) is one of the most devastating hemorrhagic strokes. SAH causes neuroinflammation and leads to both early brain injury and delayed brain injury. G‐protein‐coupled receptor 84 (GPR84), one of the orphan class‐A G protein‐coupled receptors (GPCRs), exerts pro‐inflammatory and pro‐phagocytic effects via targeting microglia in the central nervous system (CNS). This research investigated the role of GPR84 on SAH pathology via neuroinflammation.

**Methods:**

An enzyme‐linked immunosorbent assay was used for GPR84 expression in cerebrospinal fluid (CSF) samples from patients with SAH. An experimental SAH‐model mouse was established by stereotactic injection of autologous blood into the chiasmatic cisterna. The SAH model in vitro was established by exposing microglia to hemoglobin. After inhibition of GPR84 in mice by GPR84‐siRNA and GPR84‐antagonist 3, the neurological deficits were evaluated by modified Garcia test, beam balance test, and Morris water maze. Neuronal death in SAH‐model mice was evaluated by Nissl staining. GPR84, NLRP3 inflammasome, and cAMP/PKA expressions were detected by western blot and immunofluorescence.

**Results:**

GPR84 was upregulated in patients after SAH onset. The GPR84 expression in microglia increased after SAH onset, activated NLRP3 inflammasome, and promoted IL‐1β secretion. Both GPR84‐shRNA and GPR84‐antagonist 3 improved neurological deficits in SAH‐model mice. Mechanistically, GPR84 activated the NLRP3 inflammasome via the cAMP/PKA signaling pathway to aggravate neuronal injury.

**Conclusions:**

GPR84 promotes NLRP3‐mediated pyroptosis and activated NLRP3 inflammation via cAMP/PKA pathway.

## Graphical abstract

GPR84 in microglia is upregulated, activates NLRP3 inflammasomes and promotes IL‐1β secretion, which is revised by GPR84 inhibition.

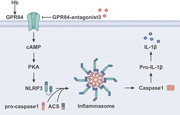

## 1 Introduction

Subarachnoid hemorrhage (SAH) is one of the most devastating hemorrhagic strokes with a high mortality and long‐term neurological morbidity (Baydoun et al. 1989; Nieuwkamp et al. 2009). The primary cause of SAH is the rupture of intracranial aneurysms (Rabinstein 2013). Complex pathophysiological mechanisms contribute to the occurrence and development of SAH. The pathophysiology procedure is divided into early brain injury (EBI) and delayed brain injury (DBI) (Macdonald 2014). EBI occurs within 72 h after SAH onset (Yamada et al. 2022) and acts as a major cause of high mortality and poor prognosis in SAH patients. During the EBI period, blood and its degradation products increase intracranial pressure, decrease cerebral blood flow, and global cerebral ischemia is aggravated. Red blood cells from ruptured aneurysms leak into the subarachnoid space, are rapidly degraded and free heme are released, thus promoting oxidative stress and activating an inflammatory cascade characterized by microglial activation, upregulation of cell adhesion molecules, recruitment of peripheral immune cells and microvascular dysfunction (Gu et al. 2021). Therefore, elucidating the multiple mechanisms of targeting EBI after SAH is crucial for preventing DBI and ameliorating the prognosis of subarachnoid hemorrhage.

The cytoplasmic polyprotein complex inflammasome is comprised of members from the Nod‐like receptor (NLR) family, as well as Pyrin and HIN domain (PYHIN) family (Fu and Wu 2023), induces pyroptosis in response to activation of multiple diverse pathogen‐associated molecular patterns (PAMPs) or damage‐associated molecular patterns (DAMPs) (Swanson et al. 2019). The NLRP3 inflammasome consists of NLRP3, ASC (the adaptor protein apoptosis‐associated speck‐like protein containing a CARD), and caspase‐1 (Zhao et al. 2021), as an innate immunomodulatory element that drives the sterile inflammatory features of many disease states (Dodd et al. 2021). The NLRP3 inflammasome oligomerizes in response to various stimuli following the onset of SAH by recruiting ASC and pro‐caspase‐1. Then activated caspase‐1 causes gasdermin D‐dependent pyroptosis (Huang et al. 2021) and converts the pro‐cytokines within the IL‐1 family into fully matured proinflammatory cytokines (Fu and Wu 2023). This excessive innate immune response results in Blood–Brain Barrier (BBB) damage and neuronal damage after SAH (C. Liu et al. 2023).

G‐protein‐coupled receptor 84 (GPR84), also named inflammation‐related G protein‐coupled receptor EX33, is one of orphan class‐A G protein‐coupled receptors (GPCRs) (Q. Zhang et al. 2022). GPR84, primarily expressed in myeloid cells such as monocytes, macrophages, and neutrophils in the periphery and microglia in the brain, constitutes a crucial component of the innate immune system (H. Liu et al. 2023). GPR84 specifically interacts with endogenous ligands such as caprylic acid, capric acid, and other medium‐chain fatty acids (MCFAs) and reduces intracellular cAMP accumulation (Qin et al. 2023). Furthermore, it impairs Camp‐induced NLRP3 polyubiquitination and degradation in macrophages, thereby enhancing the activation of the NLRP3 inflammasome (Q. Zhang et al. 2022). GPR84 activation has been verified in several studies to play a pro‐inflammatory role, functioning as a pro‐phagocytic receptor that augments the phagocytic activity of macrophages (X. Zhang et al. 2023). The involvement of GPR84 in cytokine release, phagocytosis, and status switch of lung‐resident alveolar macrophages is mediated through positive regulatory crosstalk with TLR4‐related pathways via CD14 and LBP (Yin et al. 2020), and induces neutrophils to produce reactive oxygen species (ROS) by stimulating the phosphorylation of Lyn, AKT, and ERK1/2 and the assembly of NADPH oxidase, stimulating the occurrence of lung inflammation (S. W. Wang et al. 2023). In the CNS, GPR84, as a microglial neuroinflammatory protein (Bouchard et al. 2007), mediates microglial ruffling and motility via targeting the Gi/o pathway (Wei et al. 2017). GPR84 also leads to the formation of mechanical pain and central sensitization by recruiting DOK3, which mediates the synthesis and release of downstream inflammatory factors (Gao et al. 2020). GPR84 promotes amyloid‐induced microglial proliferation to maintain dendritic homeostasis (Audoy‐Remus et al. 2015) and significantly inhibits tau hyperphosphorylation in induced pluripotent stem cell‐derived neurons in Alzheimer's disease (AD) (Qiu et al. 2024). The involvement of GPR84 in the pathology of SAH remains to be illuminated.

In the study, we extensively investigated the functional role of GPR84 in the progression of SAH through a meticulously designed series of in vitro and in vivo experiments. We hypothesized that GPR84 inhibition would inhibit the NLRP3 inflammasome, decrease IL‐1β secretion, and improve neurological outcomes in SAH‐model mice.

## 2 Materials and Methods

### 2.1 Antibodies and Molecules

The following antibodies and molecules were used in this study: GPR84 (Cat No. A12636; dilution 1:1000), Iba‐1 (Cat No. A12391; dilution 1:1000), NLRP3 (Cat No. A12694; dilution 1:1000), caspase‐1 (Cat No. A0964; dilution 1:1000), Asc (Cat No. A1170; dilution 1:1000), IL‐1β (Cat No. A16288; dilution 1:1000), cAMP (Cat No. A1640; dilution 1:1000), PKA (Cat No. A0798; dilution 1:1000), phospho‐PKA (Cat No. AP0557; dilution 1:1000), β‐actin (Cat No. AC026; dilution 1:2000) from ABclonal Technology; Goat anti‐rabbit IgG (H+L) cross‐adsorbed secondary antibody, Alexa Fluor 488 (Cat No. A‐11008; dilution 1:2000), Goat anti‐rabbit IgG (H+L) cross‐adsorbed secondary antibody, Alexa Fluor 555 (Cat No. A‐21428; dilution 1:2000) from ThermoFisher scientific.

GPR84‐shRNA lentivirus was from Genomeditech. GPR84 antagonist 3 (Cat No. HY‐151100) was from MedChemExpress. Hemoglobin (Hb) was from Sigma‐Aldrich (Cat No. H7379). Mouse IL‐1β ELISA kit (Cat No. RK04878) was from ABclonal Technology.

### 2.2 Clinical Sample Collection and Analysis

The patient was diagnosed with aneurysmal SAH by computed tomography and digital subtraction angiography and had no history of CNS disease or other organ dysfunction within 6 months. Cerebrospinal fluid (CSF) samples were collected by lumbar puncture from patients within 48 h after the onset of SAH. In contrast, CSF from the control group was obtained during spinal anesthesia prior to surgery. Samples were collected, centrifuged at 3000 r/min at 4°C for 10 min, and stored at −80°C.

### 2.3 Animal

The male adult C57BL/6 mice of clean grade were purchased from GemPharmatech (Nanjing, China) and housed under controlled temperature and humidity conditions. The mice were maintained and handled in strict accordance with guidelines set by the National Institutes of Health. All animal experiments were authorized, approved, and supervised by the Institutional Animal Care and Use Committee at Nanjing University.

### 2.4 SAH Model

The SAH model was established by stereotactic injection of autologous blood into the chiasmatic cisterna. After anesthetization, the mice were fixed and the needle was inserted through the hole into the anterior skull base. The insertion position was the anterior midline of the sagittal point, 7.5 mm from the anterior fontanel, and the direction was 45° from the coronal plane. Then 75 µL of fresh autologous blood was injected over 10 s. The control group was injected with physiological saline.

### 2.5 Experimental Design

The in vivo study was performed in three separate experiments.

#### 2.5.1 Experiment 1

Sixty‐five mice were randomly split into six distinct groups: Sham (*n* = 10) and SAH (6, 12, 24, 48, 72; *n* = 11 per group). The temporal lobes of mice (*n* = 8 for sham group; *n* = 5–6 for other groups) were collected for western blotting. Additionally, 12 mice (*n* = 3 per group) were collected for immunofluorescence.

#### 2.5.2 Experiment 2

Eighty‐three mice were randomly divided into four groups: ctr‐shRNA (*n* = 9), GPR84‐shRNA (*n* = 9), SAH + ctr‐shRNA (*n* = 8), and SAH + GPR84‐shRNA (*n* = 8). The temporal lobes of mice were collected for western blotting and immunofluorescence. Additionally, ctr‐shRNA (*n* = 8), GPR84‐shRNA (*n* = 8), SAH + ctr‐shRNA (*n* = 9), and SAH + GPR84‐shRNA (*n* = 9) were used for neurological outcomes.

#### 2.5.3 Experiment 3

Eighty‐five mice were randomly divided into four distinct groups: vehicle (*n* = 9), GPR84‐antagonist 3 (dose: 1 mg/kg) (*n* = 9), SAH + vehicle (*n* = 9), and SAH + GPR84‐antagonist 3 (*n* = 9). The temporal lobes of mice were collected for western blotting and immunofluorescence. Additionally, vehicle (*n* = 9), GPR84‐antagonist 3 (*n* = 9), SAH + vehicle (*n* = 9), and SAH + GPR84‐antagonist 3 (*n* = 9) were used for neurological outcomes.

### 2.6 Assessment of Short‐Term Neurobehavioural Outcomes

Sensorimotor function was evaluated blindly on Day 3 after SAH induction using the modified Garcia scale. Five tests were conducted, each on a scale of 0 to 3, with a maximum total score of 15.

### 2.7 Beam Balance Test

In a dimly lit chamber, position a light beam measuring 0.6 cm in width at a height of 50 cm above the ground, with one end illuminated by a lamp and the other end enclosed within a box. The caulk material was placed in the box to entice the mice to walk 30, 50, and 70 cm along the beam in turn during the training day. Before the test, the mice were exposed to light and trained 3 times a day for 3 days. When the mice walked over a distance of 80 cm, the hindlimb sliding frequency was calculated as an indicator of beam walking performance.

### 2.8 Morris Water Maze

The water maze is equally separated into four quadrants. During the acquisition phase, mice were trained four times daily for four consecutive days to find hidden platforms in fixed locations. After the acquisition phase, the platform is eliminated and the duration spent in the quadrant containing the platform, the distance moved within the quadrant of the platform, and the time passed through the platform area are measured.

### 2.9 Cell Culture

The BV2 cell line was provided by Nanjing University. The cells were cultured in a high‐glucose Dulbecco's Modified Eagle Medium (DMEM/F12) supplemented with 10% fetal bovine serum (Gibco, Cat No. A5670701) and penicillin–streptomycin (100 U/mL; ThermoFisher Scientific, Cat No. 10378016) in a humidified atmosphere containing 5% CO_2_ at 37°C. Lipofectamine 3000 (Thermo Fisher Scientific, Cat No. L3000015) was used for transfections according to the manufacturer's protocol. To establish SAH model in vitro, BV2 cells were exposed to Hb (10 µM) for 12 h.

### 2.10 Western Blotting

Brain lysates or cells were extracted and the concentration of protein was determined by the protein assay kit. The sample was loaded onto a 10% or 15% SDS‐PAGE gel. Then the protein was transferred to a polyvinylidene fluoride membrane, sealed in 5% BSA at room temperature (RT) for 1 h, and then incubated with the target antibody at 4°C overnight. Then film and two under the RT incubation resistance 2 h, using immune response protein ECL Western blotting detection reagent observation, analysis, and use ImageJ software.

### 2.11 Immunofluorescence Analysis

Brain sections were rinsed with PBS, fixed in 4% paraformaldehyde for 15 min, permeabilized with 0.3% TritonX‐100 for 30 min, and then blocked in 10% goat serum for 1 h. The brain sections were incubated with the primary antibody at 4°C overnight, followed by incubation with the corresponding secondary antibody at RT for 2 h. All sections were stained with 4, 6‐diamino‐2‐phenylindole (DAPI) and viewed under a fluorescence microscope (Carl Zeiss AG, Oberkochen, Germany).

### 2.12 Inflammatory Factors Measurement by ELISA Kits

The expressions of Inflammatory factors in CSF and culture medium were detected according to the ELISA instructions.

### 2.13 Statistical Analysis

All experiments were completely blind, assigned to hide, and randomized. Statistical analyses were performed using GraphpadPrism9.0. Data are showed as mean ± SD. One‐way analysis of variance was used for data between multiple groups. Data between two groups were analyzed by independent sample *t* test. *p* < 0.05 was statistically significant in each analysis, as follows: *p < 0.05; **p < 0.01; ***p < 0.001; ****p < 0.0001.

## 3 Results

### 3.1 Mortality and SAH Severity Scores

**FIGURE 1:**
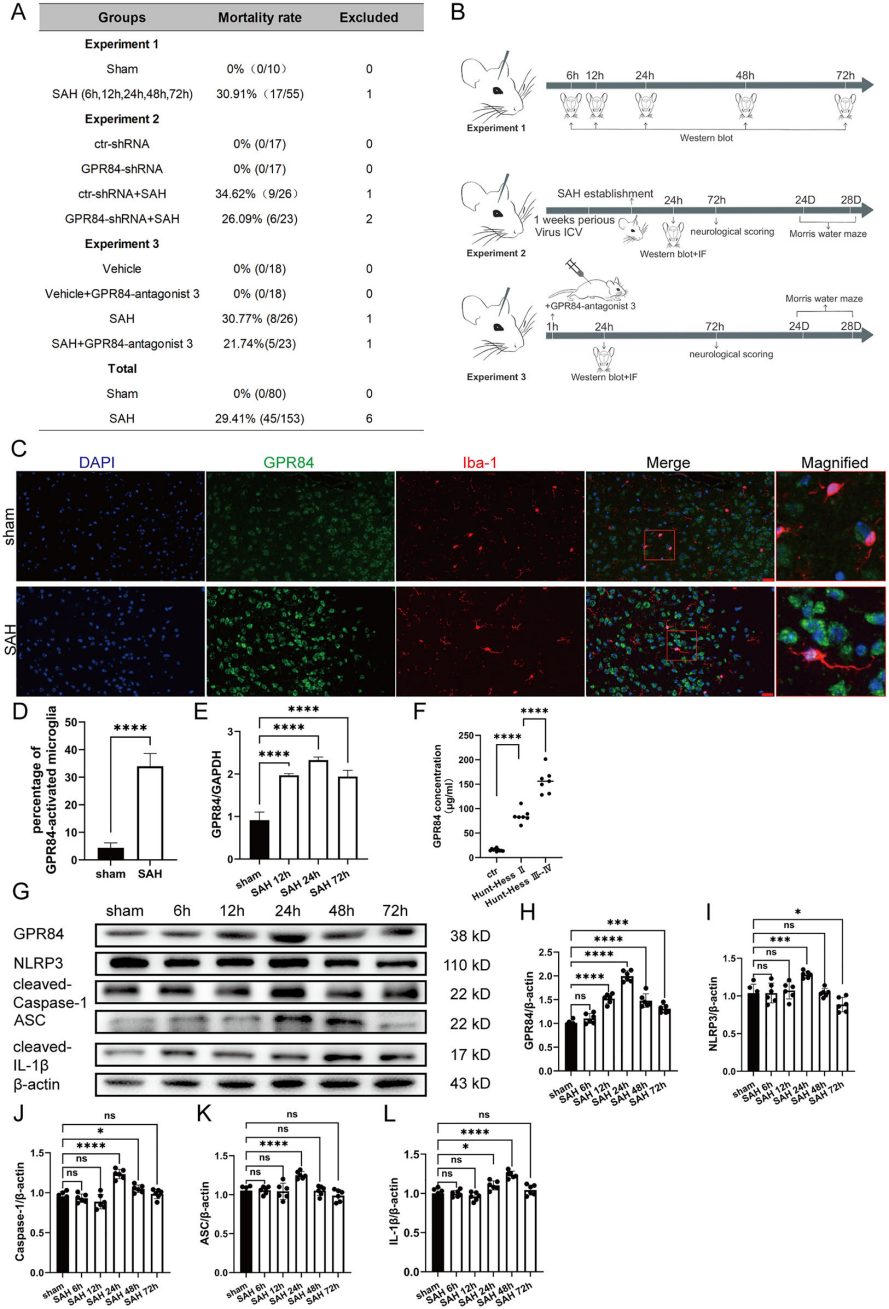
Mortality rate and time‐course expressions of different proteins after SAH. (A) The number of deaths and excluded mice from each group. (B) The time course of the three experiments in vivo. (C) Immunofluorescence staining of GPR84 (green) and Iba‐1 (red) in the sham and SAH groups. Nuclei were stained with DAPI. The right panel shows the magnification of different regions showing GPR84 in microglia. (D) Quantification of GPR84‐activated microglia in different groups; *n* = 3 mice per group. (E) The mRNA expression of GPR84 in different groups; *n* = 6 mice per group. (F) The GPR84 expression in CSF from different levels of SAH patients. (G) Representative western blot images of GPR84, ASC, NLRP3, caspase‐1, and IL‐1β in different groups; *n* = 6 per group. (H–L) Quantitative analysis of GPR84, ASC, NLRP3, caspase‐1, and IL‐1β expression. Data are presented as the mean ± SD. Data were analyzed by one‐way analysis of variance. *p < 0.05, ***p < 0.001, ****p < 0.0001.

Eighty mice were included in the sham group, and 153 were subjected to SAH induction, with six dead before SAH induction and excluded. No mortality was observed in the sham group and the total mortality rate in the SAH group was 29.41% (45/153; Figure 1A). The SAH‐treated groups were subjected to different experimental procedures (Figure 1B).

### 3.2 GPR84 and NLRP3 Inflammasomes Were Activated During EBI After SAH Onset

To confirm whether GPR84 was involved in SAH pathology, the expression of microglial GPR84 in SAH‐model mice was initially investigated. Compared to sham mice, microglial GPR84 expression increased in SAH‐model mice (*p* < 0.0001) (Figure 1C,D and Figure S1). The GPR84 mRNA level also increased and peaked at 24 h after SAH onset (*F*(3, 12) = 92.59, *p* < 0.0001) (Figure 1E). Then, a total of 24 SAH and control patients were enrolled and the clinical patient characteristics are listed in Figure S1C. The GPR84 expression from SAH and control patients was evaluated. Compared to the control group, GPR84 expression was evaluated in patients with SAH and was positively related to SAH severity (*F*(2,21) = 179.7, *p* < 0.0001) (Figure 1F). Thus, GPR84 was involved in and positively related to SAH pathology.

The protein expressions were also evaluated in brain tissues resected from mice at different times after SAH induction. The expressions of GPR84 (*F*(5,30) = 70.18, *p* < 0.0001), NLRP3 (*F*(5,30) = 10.22, *p* < 0.0001), ASC (*F*(5,30) = 10.74, *p* < 0.0001), caspase‐1 (*F*(5,30) = 23.25, *p* < 0.0001), and IL‐1β (*F*(5,30) = 19.71, *p* < 0.0001) markedly increased compared to control mice, peaked at 24 or 48 h, and decreased in mice brains after SAH (Figure 1G–L). This suggested that SAH increased GPR84 expression, activated NLRP3 inflammasome formation and subsequent pro‐inflammatory cytokine production in the mouse brain.

### 3.3 GPR84 was Required for the Inflammation Induced by SAH

**FIGURE 2:**
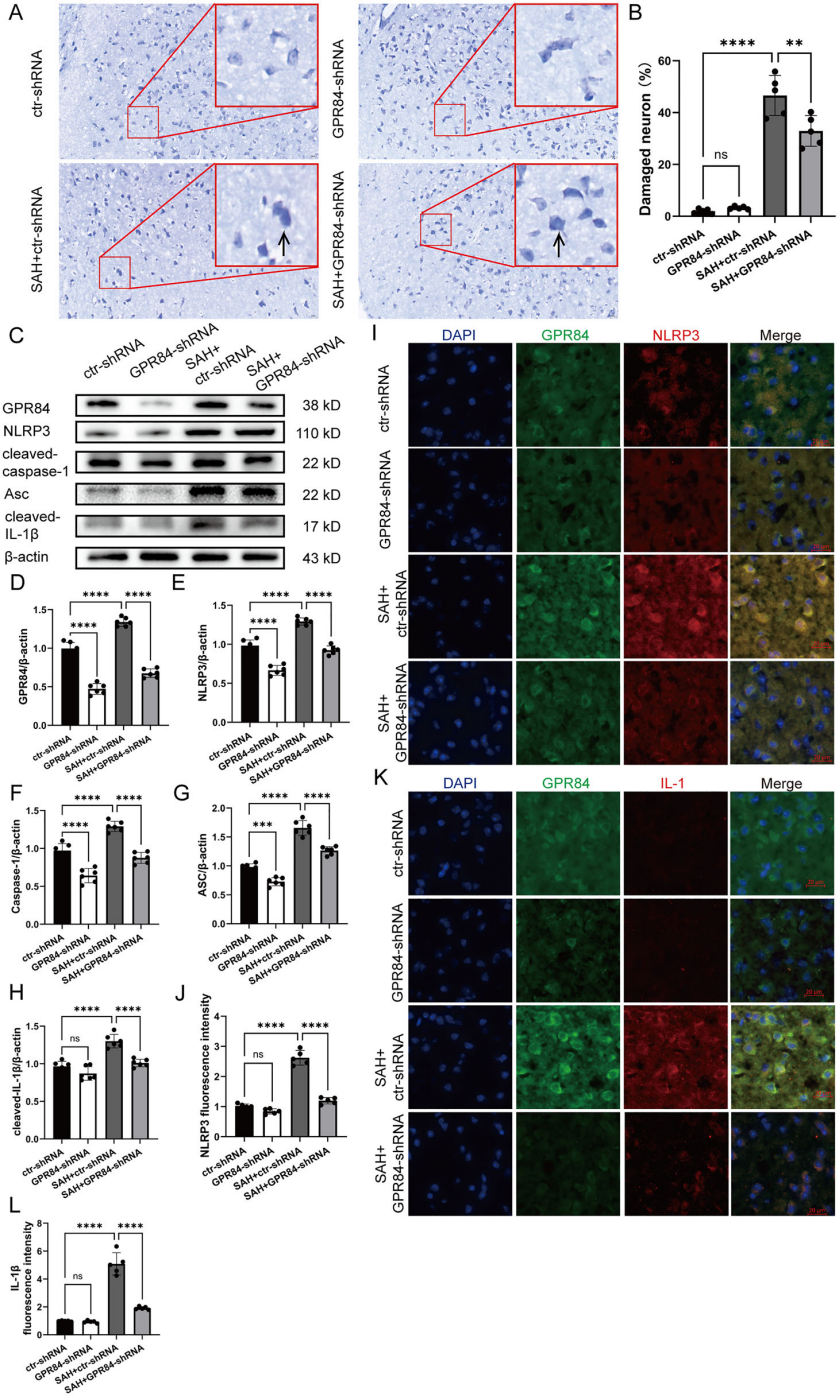
GPR84 knockdown inhibited inflammation and neuronal injury induced by SAH. (A) Representative images of Nissl staining of the temporal lobes of the ctr‐shRNA, GPR84‐shRNA, SAH + ctr‐shRNA and SAH + GPR84‐shRNA groups. The right panel shows the magnification of different regions. Arrows indicate shrunken or dead neurons. (B) Quantification of damaged neurons in the four groups; *n* = 5 mice per group. (C) Representative western blot images of GPR84, ASC, NLRP3, caspase‐1, and IL‐1β expressions from ctr‐shRNA, GPR84‐shRNA, SAH + ctr‐shRNA and SAH + GPR84‐shRNA groups; *n* = 6 per group. (D–H) Quantitative analysis of GPR84, ASC, NLRP3, caspase‐1, and IL‐1β expressions from different groups. (I) Immunofluorescence staining for GPR84 (green) and NLRP3 (red) in the ctr‐shRNA, GPR84‐shRNA, SAH + ctr‐shRNA and SAH + GPR84‐shRNA groups. (J) Quantification of NLRP3 immunofluorescence intensity in the four groups; *n* = 3 mice per group. (K) Immunofluorescence staining for GPR84 (green) and IL‐1β (red) in the ctr‐shRNA, GPR84‐shRNA, SAH + ctr‐shRNA and SAH + GPR84‐shRNA groups. Nuclei were stained with DAPI. (L) Quantification of IL‐1β immunofluorescence intensity in the four groups; *n* = 3 mice per group. **p < 0.01, ***p < 0.001, ****p < 0.0001.

To determine whether GPR84 exerts proinflammatory effects, we used a GPR84‐shRNA lentivirus to downregulate GPR84 expression. Nissl staining indicated that SAH caused neuronal death, and GPR84‐shRNA attenuated neuronal injury (*F*(3,16) = 103.1, *p* < 0.0001) (Figure 2A,B). Western blotting showed SAH upregulated NLRP3 (*F*(3,20) = 103.1, *p* < 0.0001), ASC (*F*(3,20) = 131.1, *p* < 0.0001), caspase‐1 (*F*(3,20) = 63.48, *p* < 0.0001), and IL‐1β (*F*(3,20) = 35.28, *p* < 0.0001) expressions, and inhibition of GPR84 inhibited NLRP3 inflammasome formation (Figure 2C–H). Immunofluorescence staining also showed SAH upregulated GPR84, NLRP3 (*F*(3,16) = 161.7, *p* < 0.0001), and IL‐1β (*F*(3,16) = 115.9, *p* < 0.0001) expression, which was reversed by GPR84‐shRNA (Figure 2I–L). This suggested GPR84 inhibition reversed NLRP3 inflammasome and attenuated neuronal injury after SAH onset.

### 3.4 GPR84 Inhibition Improves Short‐Term Neurobehavior and Rescues Cognitive Deficits in SAH‐Model Mice

**FIGURE 3:**
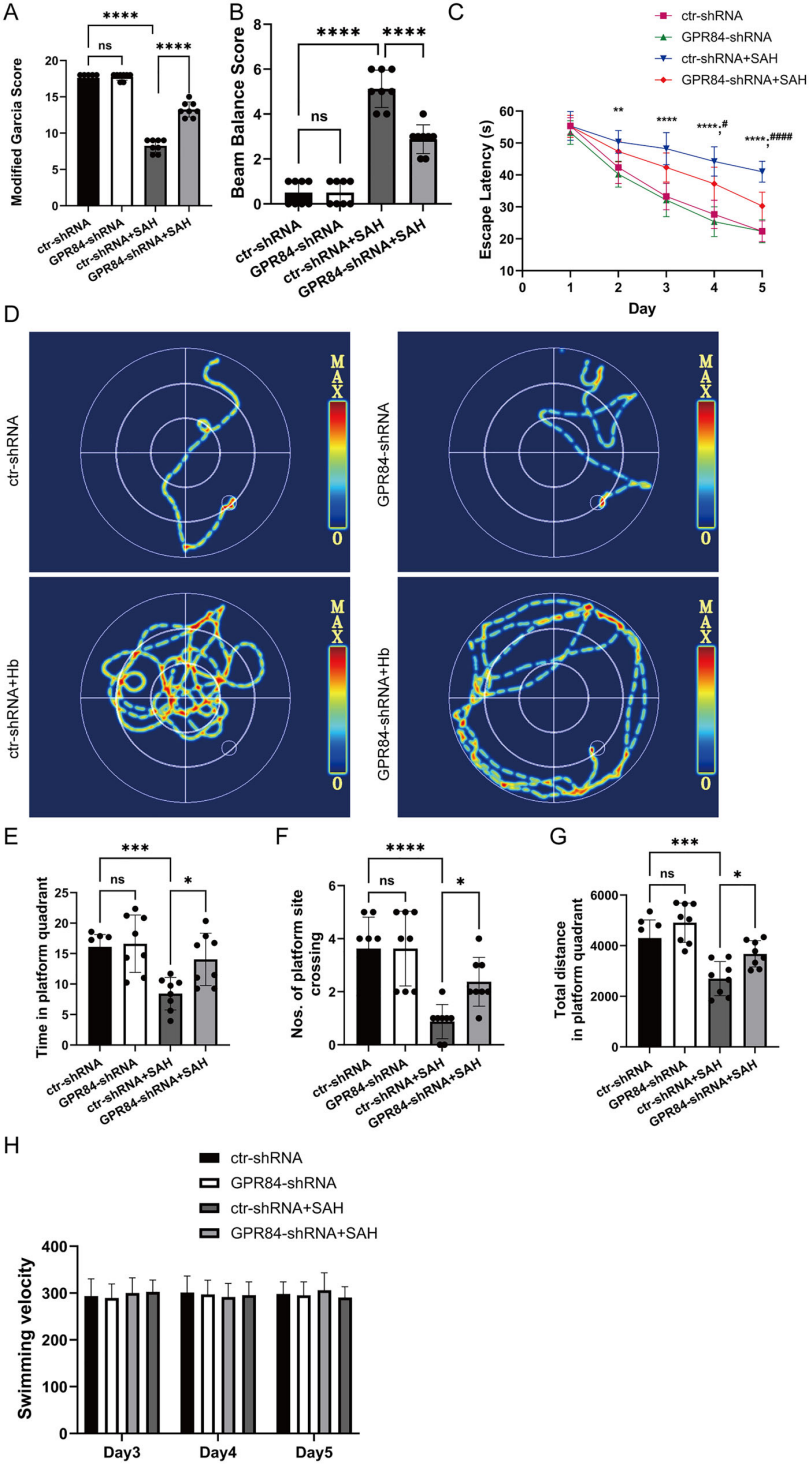
GPR84 knockdown improves short‐term neurobehavior, and rescues cognitive deficits in subarachnoid hemorrhage (SAH)‐model mice. Mice were intracerebrally injected with GPR84‐shRNA lentivirus for 1 week and underwent SAH procedure. The mice were subjected to short‐term neurobehavioral evaluation (A and B) (*n* = 6 per group), and Morris water maze (C–H) (*n* = 8–9 per group). (A and B) Modified Garcia scores and beam balance scores. (C) Escape latency. (D) Representative images of the path along which the mice swam to find the platform. (E) Number of mice that crossed the platform after retrieval. (F) Time taken by mice to swim in the target platform quadrant after platform retrieval. (G) Total distance in the target platform quadrant after platform retrieval. (H) Swimming speed. Data are presented as the mean ± SD. Data were analyzed using a one‐way analysis of variance. *p < 0.05, ***p < 0.001, ****p < 0.0001.

To elucidate the involvement of GPR84 in cognitive impairments following SAH, we examined whether GPR84 inhibition could attenuate short‐term neurological deficits in an SAH‐model mouse. The modified Garcia (*F*(3,28) = 276.5, *p* < 0.0001) and beam balance scores (*F*(3,28) = 93.93, *p* < 0.0001) indicated neurological deficits in the SAH group compared to the sham group (Figure 3A,B). However, the intracerebral injection of GPR84 lentivirus significantly improved neurological scores compared to those in the SAH group (Figure 3A,B). In the Morris water maze test, escape latencies and swimming distances in consecutive trials increased in SAH mice compared with those in the control group, indicating SAH mice impaired learning. In contrast, GPR84 inhibition significantly ameliorated these changes in performance, with shorter escape latencies and swimming distances (all *p* < 0.05, *n* = 8) (Figure 3C,D). In the probe quadrant trial, SAH mice spent less time in the target quadrant than those in the sham group (*F*(3,28) = 8.74, *p* < 0.001) (Figure 3E). Moreover, GPR84 lentivirus significantly increased the time spent in the target quadrant and the total distance covered within the target quadrant (*F*(3,28) = 15.43, *p* < 0.0001), and mice swam over the target site more frequently compared to the SAH mice (*F*(3,28) = 11.77, *p* < 0.0001) (Figure 3E–G). The differences among these groups of mice were not attributed to their distinct swimming capabilities, as the swimming speeds in these groups of mice were comparable (all *p* > 0.05, *n* = 8) (Figure 3H). These results indicated that GPR84 inhibition could improve both short‐ and long‐term neurological behaviors in mice with SAH.

### 3.5 GPR84 accelerated Neuroinflammation via cAMP/PKA Signaling in Vitro

**FIGURE 4:**
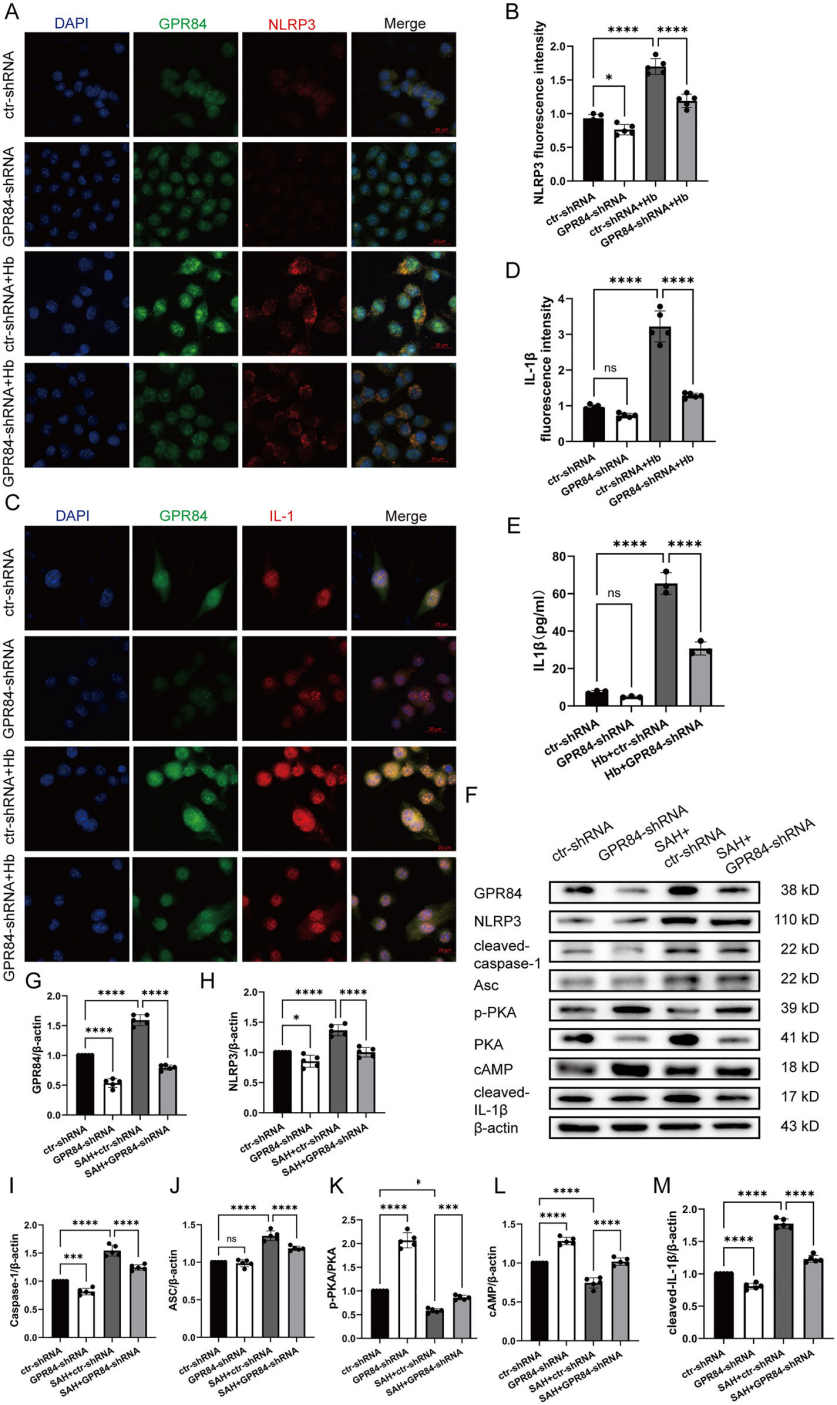
GPR84 knockdown inhibited NLRP3 inflammasomes and IL‐1β secretion in vitro. (A) Immunofluorescence staining for GPR84 (green) and NLRP3 (red) in the ctr‐shRNA, GPR84‐shRNA, SAH + ctr‐shRNA and SAH+GPR84‐shRNA groups. (B) Quantification of NLRP3 immunofluorescence intensity in the four groups; *n* = 6 sections per group. (C) Immunofluorescence staining for GPR84 (green) and IL‐1β (red) in the ctr‐shRNA, GPR84‐shRNA, SAH + ctr‐shRNA and SAH+GPR84‐shRNA groups. Nuclei were stained with DAPI. (D) Quantification of IL‐1β immunofluorescence intensity in the four groups; *n* = 6 sections per group. (E) IL‐1β concentration in the cell culture media of the four groups. (F) Representative western blot images of GPR84, cAMP, p‐PKA, PKA, ASC, NLRP3, caspase‐1, and IL‐1β expressions from ctr‐shRNA, GPR84‐shRNA, SAH + ctr‐shRNA and SAH+GPR84‐shRNA groups; *n* = 5 per group. (G–M) Quantitative analysis of GPR84, cAMP, p‐PKA/PKA, ASC, NLRP3, caspase‐1, and IL‐1β expressions from different groups. *p < 0.05, ***p < 0.001, ****p < 0.0001.

To illustrate the mechanism of GPR84 activating neuroinflammation, BV2 cells were transfected with GPR84‐shRNA for 48 h and treated with hemoglobin (Hb) for 12 h. Hb increased both NLRP3 (*F*(3,16) = 102.8, *p* < 0.0001) and IL‐1β (*F*(3,16) = 128, *p* < 0.0001) immunofluorescent intensity accompanied by GPR84 up‐expression (Figure 4A–D). Hb induced an increase in extracellular IL‐1β level of culture medium compared to the control, and GPR84‐shRNA reversed the upregulation of IL‐1β level (*F*(3,8) = 202.1, *p* < 0.0001) (Figure 4E). Western blotting also showed Hb upregulated GPR84 (*F*(3,16) = 260.9, *p* < 0.0001), NLRP3 (*F*(3,16) = 38.93, *p* < 0.0001), ASC (*F*(3,16) = 81.71, *p* < 0.0001), Caspase‐1 (*F*(3,16) = 132.1, *p* < 0.0001), IL‐1β (*F*(3,16) = 327.9, *p* < 0.0001) expressions and inhibited cAMP (*F*(3,16) = 107.7, *p* < 0.0001), p‐PKA (*F*(3,16) = 285, *p* < 0.0001) expressions (Figure 4F–M). GPR84‐shRNA increased cAMP and p‐PKA expressions, as well as inhibiting NLRP3 inflammasomes.

### 3.6 GPR84‐antagonist 3 Inhibited NLRP3 Inflammasomes and Improved Neurological Deficits in Vivo

**FIGURE 5:**
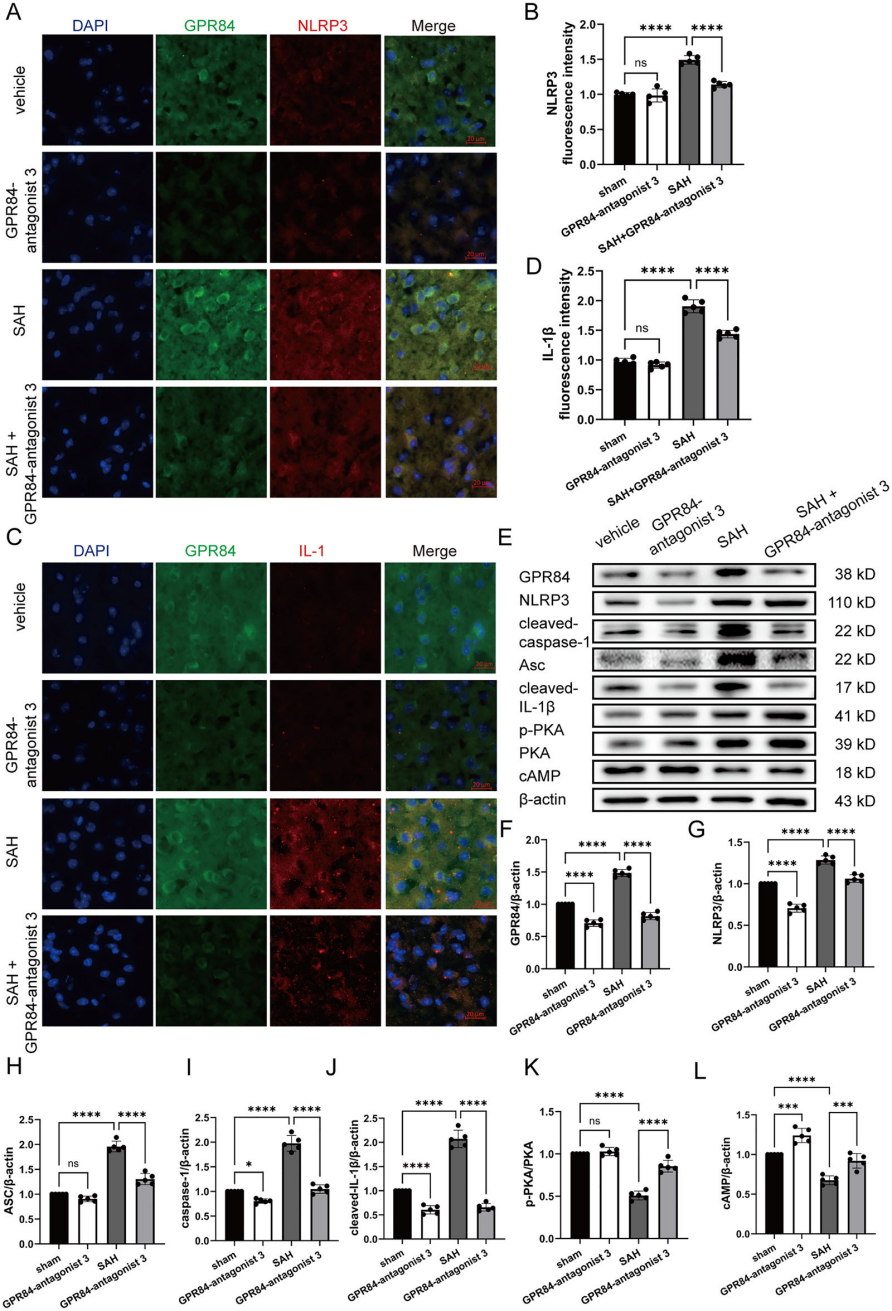
GPR84‐antagonist 3 inhibited NLRP3‐mediated neuroinflammation in vivo. (A) Immunofluorescence staining for GPR84 (green) and NLRP3 (red) in the sham, GPR84‐antagonist 3, SAH and SAH+GPR84‐antagonist 3 groups. (B) Quantification of NLRP3 immunofluorescence intensity in the four groups; *n* = 3 mice per group. (C) Immunofluorescence staining for GPR84 (Green) and IL‐1β (red) in the sham, GPR84‐antagonist 3, SAH and SAH + GPR84‐antagonist 3 groups. Nuclei were stained with DAPI. (D) Quantification of IL‐1β immunofluorescence intensity in the four groups; *n* = 3 mice per group. (E) Representative western blot images of GPR84, cAMP, p‐PKA, PKA, ASC, NLRP3, caspase‐1, and IL‐1β expressions from sham, GPR84‐antagonist 3, SAH and SAH+GPR84‐antagonist 3 groups; *n* = 6 mice per group. (F–L) Quantitative analysis of GPR84, cAMP, p‐PKA/PKA, ASC, NLRP3, caspase‐1, and IL‐1β expressions from different groups. *p < 0.05, ***p < 0.001, ****p < 0.0001.

**FIGURE 6:**
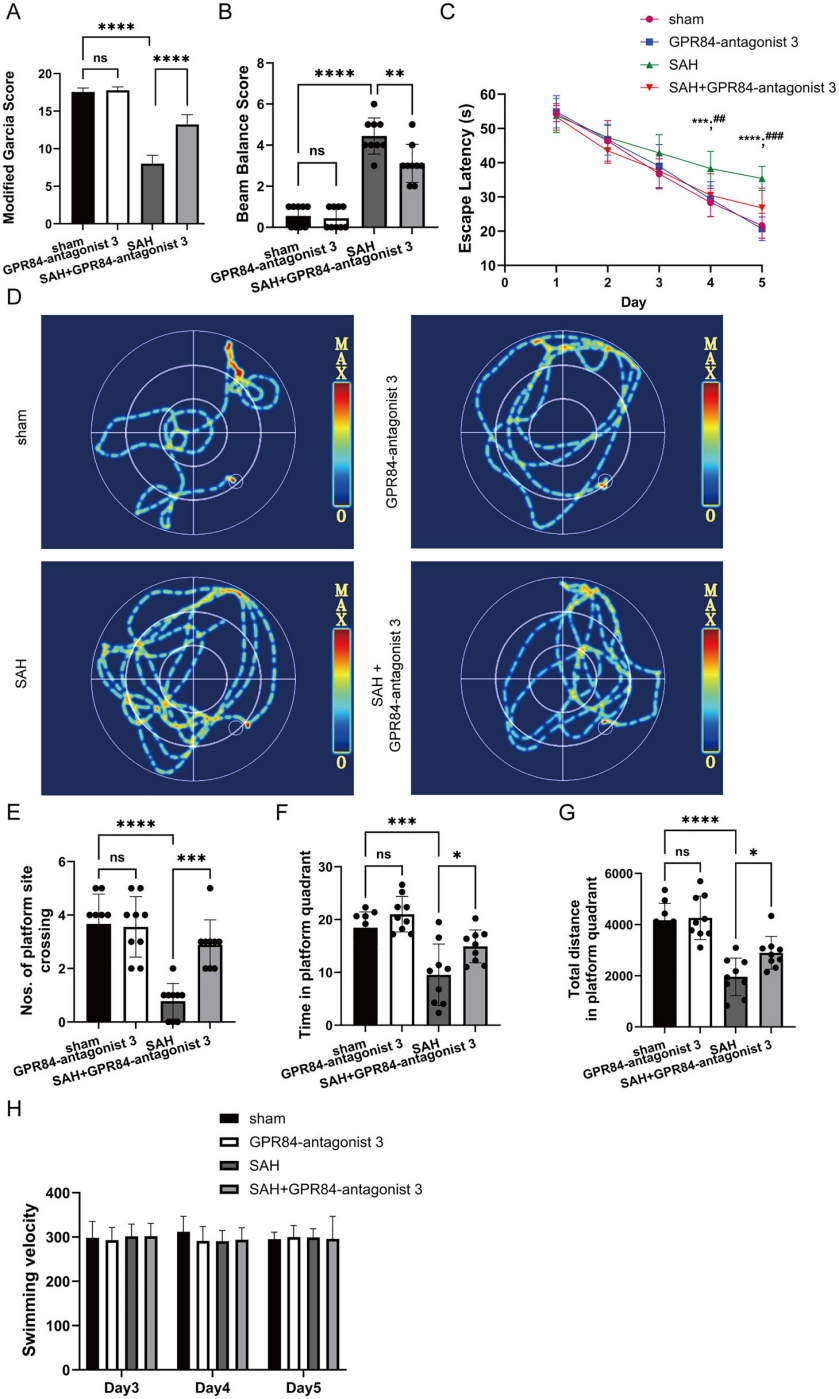
GPR84‐antagonist 3 improved short‐term neurobehavior, and rescues cognitive deficits in subarachnoid hemorrhage (SAH)‐model mice. Sham and SAH mice were intravenously injected with GPR84‐antagonist 3 once. The mice were subjected to short‐term neurobehavioral evaluation (A and B) (*n* = 6 per group), and Morris water maze (C–H) (*n* = 8–9 per group). (A and B) Modified Garcia scores and beam balance scores. (C) Escape latency. (D) Representative images of the path along which the mice swam to find the platform. (E) Number of mice that crossed the platform after retrieval. (F) Time taken by mice to swim in the target platform quadrant after platform retrieval. (G) Total distance travelled by mice in the target platform quadrant after platform retrieval. (H) Swimming speed. Data are presented as the mean ± SD. Data were analyzed using a one‐way analysis of variance. *p < 0.05, **p < 0.01, ***p < 0.001, ****p < 0.0001.

To confirm the therapeutic effect of GPR84 after SAH onset, GPR84‐antagonist 3 was intravenously injected once at a dose of 1 mg/kg after SAH onset. Immunofluorescence staining revealed GPR84‐antagonist 3 decreased GPR84, NLRP3 (*F*(3,16) = 71.91, *p* < 0.0001), and IL‐1β (*F*(3,16) = 192.7, *p* < 0.0001) expressions (Figure 5A–D). Western blotting also indicated GPR84 (*F*(3,16) = 270.4, *p* < 0.0001) expression, and NLRP3 inflammasomes decreased accompanied with cAMP (*F*(3,16) = 55.92, *p* < 0.0001) and *p*‐PKA (*F*(3,16) = 113.3, *p* < 0.0001) upregulation after GPR84‐antagonist 3 treatment (Figure 5E–L). Then both the short‐term and long‐term neurological deficits were determined after GPR84‐antagonist 3 treatment. Both Modified Garcia Score (*F*(3,32) = 222.8, *p* < 0.0001) and beam balance scores (*F*(3,32) = 63.65, *p* < 0.0001) were improved after GPR84‐antagonist 3 treatment (Figure 6A,B). In the Morris water maze test, GPR84‐antagonist 3 improved impaired learning, as indicated by decreased escape latencies (Day 3: *p* < 0.01, *n* = 9; Day 4: *p* < 0.001, *n* = 9) and swimming distances in consecutive trials compared with those in the SAH group (Figure 6C,D). Furthermore, GPR84‐antagonist 3 treatment significantly enhanced the time spent in the target quadrant (*F*(3,32) = 13.97, *p* < 0.0001) and the total distance traveled in the target quadrant (*F*(3,32) = 22.88, *p* < 0.0001), and mice swam over the target site (*F*(3,32) = 16.89, *p* < 0.0001) more frequently compared to observations in the SAH group (Figure 6E–G). The differences among these groups of mice were also not attributed to their distinct swimming capabilities (all *p* > 0.05, *n* = 9) (Figure 6H).

## 4 Discussion

In the present study, we demonstrated that GPR84 was activated in microglia and accelerated NLRP3 inflammasome formation after SAH onset. GPR84‐shRNA increased cAMP/PKA signaling and inhibited NLRP3 inflammasomes and IL‐1β maturation in vivo and in vitro. GPR84 inhibition by GPR84‐shRNA or GPR84‐antagonist 3 improved both short‐ and long‐term outcomes in SAH model mice.

The pathophysiology of SAH is complex, and recent studies indicated that neuroinflammation exerts a fundamental role in the pathogenesis of EBI following SAH onset (Coulibaly and Provencio 2020; C. Liu et al. 2023). Microglia activation exerts a remarkable effect on the inflammatory response (Chen et al. 2022). Our results suggested that the inflammatory response followed by microglia activation and cytokine up‐expression was developed in SAH‐model mice. Excessive inflammatory response attributed to neuronal injury after SAH. Therefore, attenuating the excessive inflammation is fundamental for decreasing neuronal death. Pyroptosis is viewed as an inflammatory‐mediated programmed cell death, and the landmark is the plasma membrane disruption. The typical pyroptosis pathway is activated by NLRP3 inflammasomes (Xue et al. 2019). NLRP3 manifests as a sensor and undergoes self‐oligomerization under a variety of stimuli. Oligomerized NLRP3 recruits ASC and pro‐caspase‐1 to assemble the NLRP3‐ASC‐caspase‐1 protein complex, which is commonly referred to as inflammasomes (Lu et al. 2014). The formation of the NLRP3 inflammasome triggers the cleavage of pro‐caspase‐1, maturation of pro‐IL‐1β, and subsequent release. The activation of caspase‐1 also leads to the cleavage of gasdermin D (GSDMD) and the subsequent release of its N‐terminal domain. These domains are transported to the cell membranes and form pores to accelerate the release of cytokines (He et al. 2015; Shi et al. 2015). Our study found the expression of IL‐1β increased in the CSF of SAH patients. The expressions of NLRP3 and IL‐1β were also increased in the temporal lobe of SAH‐model mice and BV2 cells treated with Hb. Activated NLRP3 could promote both short‐ and long‐term neurological impairments in SAH‐model mice. These results indicated NLRP3‐mediated pyroptosis was activated after SAH onset and may serve as a therapeutic target for EBI after SAH.

GPCRs represent the most abundant membrane protein family that enables them to detect environmental changes and activate the appropriate intracellular signaling and cellular functions (Forsman et al. 2024). GPR84, also known as FFAR2, is proven to promote proinflammatory response and cytokine release in several diseases (M. Wang et al. 2019; S. W. Wang et al. 2023; Q. Zhang et al. 2022). In AD, microglial GPR84 is upregulated in APP/PS1 transgenic mice. And GPR84 knockdown promotes dendritic degeneration and cognitive decline (Audoy‐Remus et al. 2015). In several depressive disorders, low‐dose LPS attenuates overactive microglia and severe neuroinflammation via GPR84, which attributes inflammation‐induced depressive‐like behavior in mice (Yu et al. 2023). However, the mechanism of GPR84 regulating pyroptosis in EBI after SAH remains unknown. In our study, the expression of GPR84 increased significantly, peaked at 24 h, and decreased during EBI in vivo. GPR84 knockdown inhibited NLRP3 inflammasome formation and attenuated neuronal death. GPR84‐antagonist 3 administration reversed NLRP3 inflammasome and cytokine expression in vivo. Both GPR84‐shRNA and GPR84‐antagonist 3 improved neurological deficits in SAH‐model mice.

While GPR84‐activating pyroptosis has already been confirmed, the precise mechanisms remain unclear. Several studies have demonstrated the cAMP/PKA signaling pathway is involved in regulating several physiological processes, for example, apoptosis and synaptic plasticity (Waltereit and Weller 2003). Neuroinflammation can also be suppressed by enhancing cAMP/PKA signaling pathway in both the neurodegeneration‐model and the LPS‐induced model (Li et al. 2021; Park et al. 2016). The cAMP/PKA signaling pathway is also involved in inhibiting the NLRP3 inflammasomes via ubiquitination and degradation (Hu et al. 2021; Yan et al. 2015). These studies have proven cAMP reverses NLRP3 inflammasome via confining its assembly. However, activated GPR84 inhibits adenylate cyclase activity, thus leading to reduced cAMP production (Q. Zhang et al. 2022). In our study, GPR84 activates NLRP3 inflammasomes via inhibiting cAMP/PKA signaling pathway. Therefore, our study provided the initial validation that GPR84 regulates NLRP3 inflammasome‐induced pyroptosis via the cAMP/PKA signaling pathway.

The study, however, still has certain limitations. Initially, we exclusively elucidated the involvement of GPR84 in microglial response following SAH. However, there exist various immune cell infiltrations and actions after SAH onset; the role of GPR84 in other immune cells needs further investigation. Second, to further clarify the impact of GPR84 on NLRP3‐mediated pyroptosis, it is suggested to employ microglia‐specific GPR84 knockout mice for validation of its effects on NLRP3‐mediated pyroptosis and subsequent neurological deficits following SAH onset. Third, the expression of GPR84 in non‐ aneurysmal SAH and the mechanism of GPR84 expression changes during EBI remain unknown and needs further investigation.

## 5 Conclusion

In summary, the current study verified the increased GPR84 expression in microglia after SAH onset. GPR84 inhibition alleviated both NLRP3 inflammasome formation and neuronal injury via the cAMP/PKA pathway. The GPR84‐antagonist 3 improved neurological deficits in SAH model mice. These results indicate that GPR84 is a potential target for the clinical treatment of EBI.

## Author Contributions

**Kun Jiang**: formal analysis, validation, project administration, conceptualization, writing–original draft, methodology. **Yan Zou**: writing–original draft, methodology, formal analysis, project administration, validation, conceptualization. **Yue Song**: software, data curation, visualization. **Long'jiang Zhou**: formal analysis, data curation, project administration, methodology. **Bing'tao Zhang**: software, visualization, validation. **Xiao'ming Zhou**: methodology, supervision; resources; funding acquisition; investigation. **Xin Zhang**: conceptualization, methodology, software, funding acquisition, writing‐review and editing, supervision, resources, validation.

## Ethics Statement

This study was approved by the Ethics Committee of Jinling Hospital and was conducted in accordance with the principles of Good Clinical Practice and Declaration of Helsinki. All patients in this study provided signed informed consent. All animal experiments were authorized, approved and supervised by the Institutional Animal Care and Use Committee at Nanjing University. The animals were raised and used in strict accordance with the National Institutes of Health guidelines.

## Conflicts of Interest

The authors declare no conflicts of interest.

### Peer Review

The peer review history for this article is available at https://publons.com/publon/10.1002/brb3.70379.

## Supporting information




**Figure S1**. GPR84 expression only alters in microglia after in subarachnoid hemorrhage (SAH). (A) Double immunofluorescence staining for GPR84 (green), and NeuN (red), GFAP (red), and Iba‐1 (red) in sham and SAH model mice. Scale bar = 50 µm. (B) Quantitative analysis of GPR40 expression in neurons, astrocytes, and microglia in temporal lobes (*n* = 5). (C) Clinical Characteristics of patients enrolled in this study. **p < 0.01.

**Figure S2**. GPR84 overexpression increased NLRP3 inflammasomes and IL‐1β secretion in vitro. (A) Immunofluorescence staining for GPR84 (green) and NLRP3 (red) in the ctr‐overexpression (ctr‐oe), GPR84‐oe, SAH + ctr‐oe and SAH+GPR84‐oe groups. (B) Quantification of NLRP3 immunofluorescence intensity in the four groups; *n* = 5 sections per group. (C) Immunofluorescence staining for GPR84 (green) and IL‐1β (red) in the ctr‐oe, GPR84‐oe, SAH + ctr‐oe and SAH + GPR84‐oe groups. Nuclei were stained with DAPI. (D) Quantification of IL‐1β immunofluorescence intensity in the four groups; *n* = 5 sections per group. (E) Representative western blot images of GPR84, cAMP, p‐PKA, PKA, ASC, NLRP3, caspase‐1, and IL‐1β expressions from ctr‐shRNA, GPR84‐shRNA, SAH + ctr‐shRNA, and SAH + GPR84‐shRNA groups; *n* = 5 per group. (F–L) Quantitative analysis of GPR84, cAMP, p‐PKA/PKA, ASC, NLRP3, caspase‐1, and IL‐1β expressions from different groups. *p < 0.05, **p < 0.01, ***p < 0.001, ****p < 0.0001.

## Data Availability

The data that support the findings of this study are available from the corresponding author upon reasonable request.
