# Supplementary figures and images for: G‐Protein‐Coupled Receptor 84 Aggravates Early Brain Injury via Microglial NLRP3‐ASC Inflammasome After Subarachnoid Hemorrhage
Source: Brain Behav. 2025 Apr 9;15(4):e70379. doi: 10.1002/brb3.70379 (PMC11979352; doi:10.1002/brb3.70379)

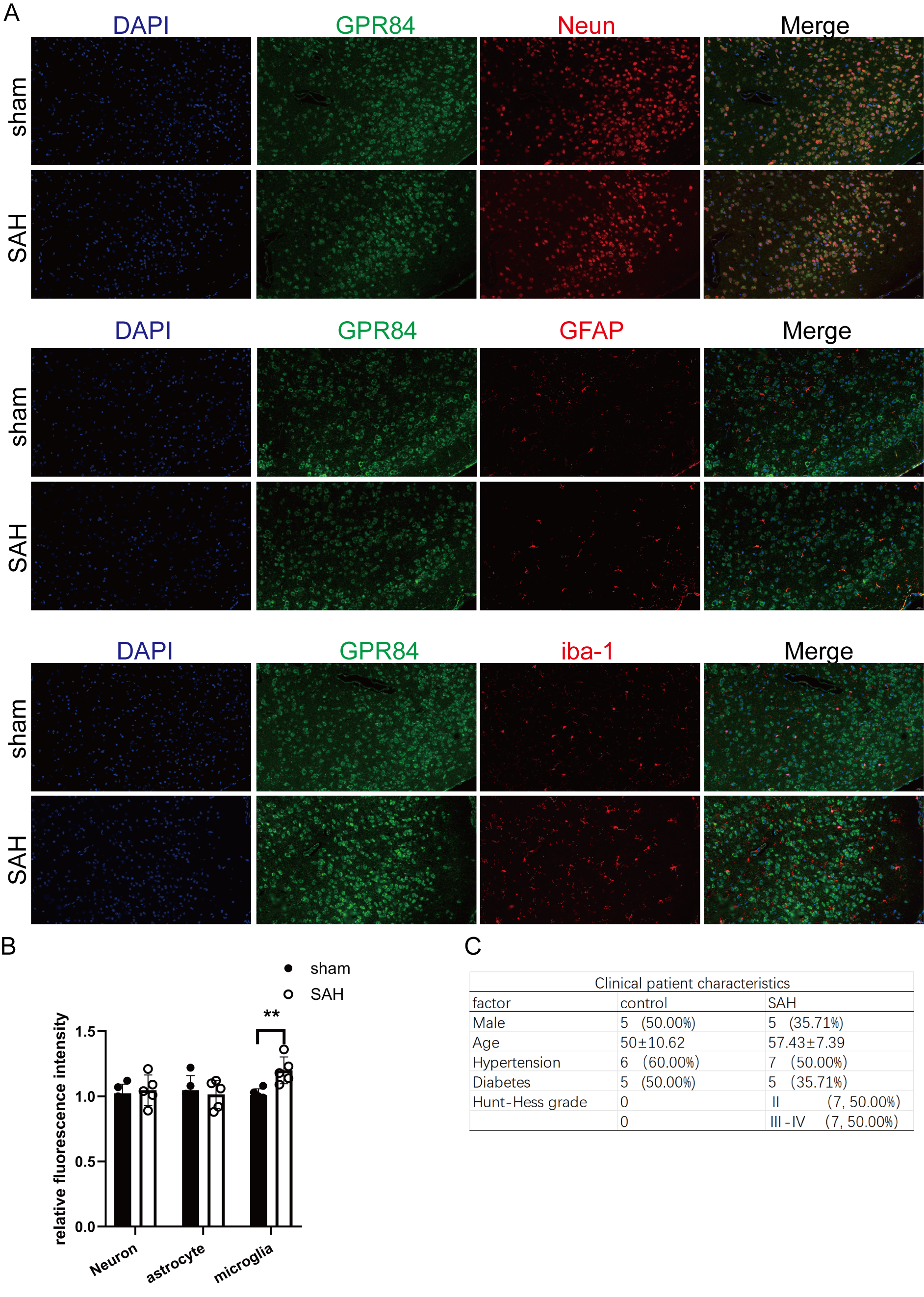

Supplement: Supplementary file 1 — Figure S1. GPR84 expression only alters in microglia after in subarachnoid hemorrhage (SAH). (A) Double immunofluorescence staining for GPR84 (green), and NeuN (red), GFAP (red), and Iba‐1 (red) in sham and SAH model mice. Scale bar = 50 µm. (B) Quantitative analysis of GPR40 expression in neurons, astrocytes, and microglia in temporal lobes (n = 5). (C) Clinical Characteristics of patients enrolled in this study. **p < 0.01. [file BRB3-15-e70379-s001.tif]

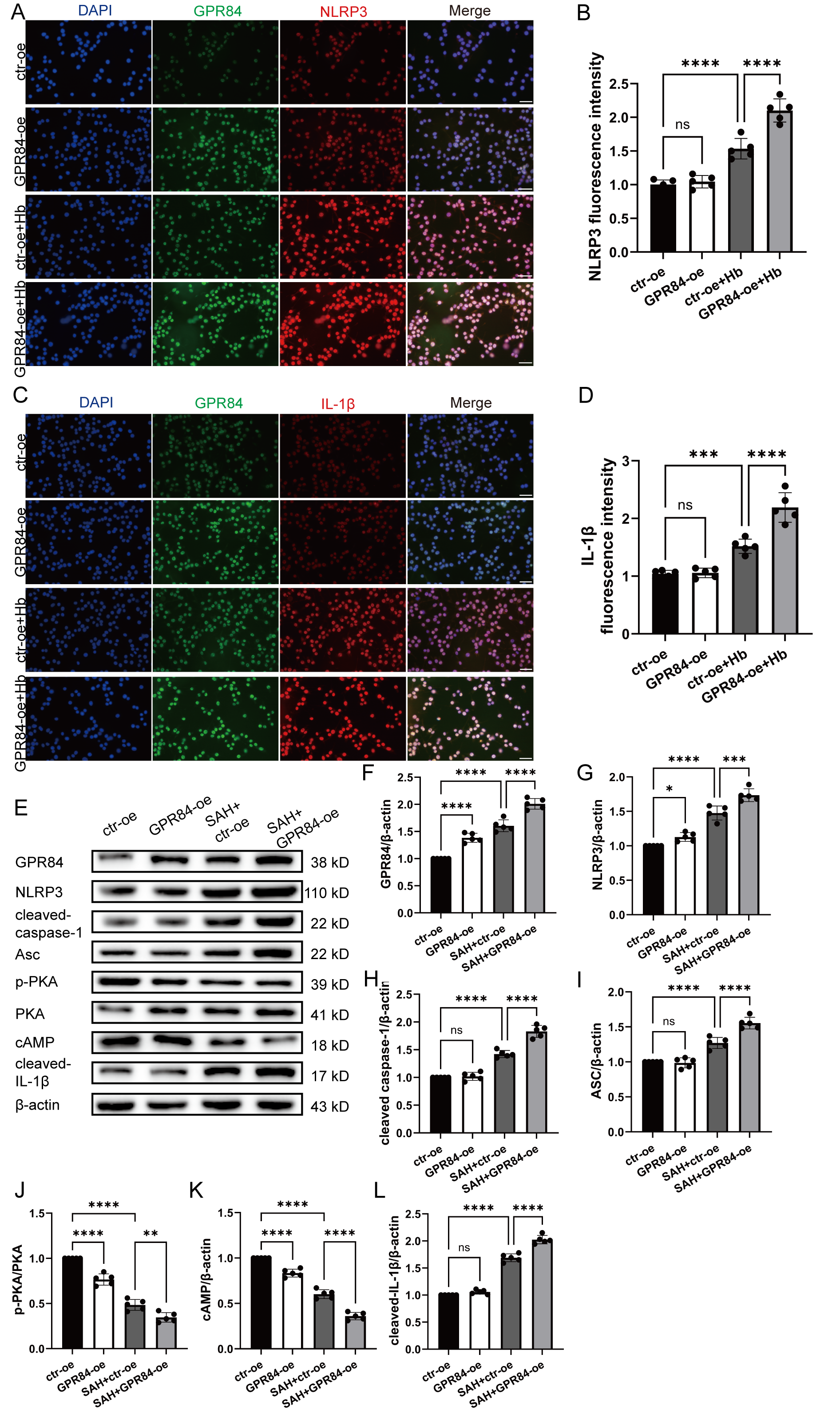

Supplement: Supplementary file 2 — Figure S2. GPR84 overexpression increased NLRP3 inflammasomes and IL‐1β secretion in vitro. (A) Immunofluorescence staining for GPR84 (green) and NLRP3 (red) in the ctr‐overexpression (ctr‐oe), GPR84‐oe, SAH + ctr‐oe and SAH+GPR84‐oe groups. (B) Quantification of NLRP3 immunofluorescence intensity in the four groups; n = 5 sections per group. (C) Immunofluorescence staining for GPR84 (green) and IL‐1β (red) in the ctr‐oe, GPR84‐oe, SAH + ctr‐oe and SAH + GPR84‐oe groups. Nuclei were stained with DAPI. (D) Quantification of IL‐1β immunofluorescence intensity in the four groups; n = 5 sections per group. (E) Representative western blot images of GPR84, cAMP, p‐PKA, PKA, ASC, NLRP3, caspase‐1, and IL‐1β expressions from ctr‐shRNA, GPR84‐shRNA, SAH + ctr‐shRNA, and SAH + GPR84‐shRNA groups; n = 5 per group. (F–L) Quantitative analysis of GPR84, cAMP, p‐PKA/PKA, ASC, NLRP3, caspase‐1, and IL‐1β expressions from different groups. *p < 0.05, **p < 0.01, ***p < 0.001, ****p < 0.0001. [file BRB3-15-e70379-s002.tif]
